# Supplementary material for: The Emotion Regulation Questionnaire: Psychometric Properties and Prediction of Posttraumatic Consequences during the COVID-19 Pandemic in Chilean Adults
Source: Int J Environ Res Public Health. 2023 Feb 16;20(4):3452. doi: 10.3390/ijerph20043452 (PMC9967314; doi:10.3390/ijerph20043452)
Supplement: Supplementary file 1 [file ijerph-20-03452-s001.zip › ijerph-2165214-supplementary.pdf]

ANNEX

EMOTION REGULATION QUESTIONNAIRE (ERQ)

Instructions and Items:

We would like to ask you some questions about your emotional life, in particular, how you control (that is, regulate and manage) your emotions. The questions below involve two distinct aspects of your emotional life. One is your emotional experience, or what you feel like inside. The other is your emotional expression, or how you show your emotions in the way you talk, gesture, or behave. Although some of the following questions may seem similar to one another, they differ in important ways.

For each item, please answer using the following scale:

1	2	3	4	5	6	7
strongly disagree [Totalmente en desacuerdo]			Neutral [Indiferente]			strongly agree (Totalmente de acuerdo]

1. When I want to feel more positive emotion (such as joy or amusement), I change what I'm thinking about [Cuando quiero sentir emociones más agradables (como alegría o felicidad), cambio lo que estoy pensando]

2. I keep my emotions to myself [Me guardo mis emociones para mí, no las expreso]

3. When I want to feel less negative emotion (such as sadness or anger), I change what I'm thinking about [Cuando NO quiero sentir emociones desagradables (como tristeza o enojo), cambio lo que estoy pensando]

4. When I am feeling positive emotions, I am careful not to express them [Cuando estoy sintiendo emociones agradables, me cuido de NO expresarlas]

5. When I'm faced with a stressful situation, I make myself think about it in a way that helps me stay calm [Cuando me enfrento a una situación estresante, me esfuerzo en pensar sobre la situación de una manera que me ayude a mantener la calma]

6. I control my emotions by not expressing them [Controlo mis emociones NO expresándolas]

7. When I want to feel more positive emotion, I change the way I'm thinking about the situation [Cuando quiero sentir emociones más agradables, cambio la manera en que veo la situación]

8. I control my emotions by changing the way I think about the situation I'm in [Controlo mis emociones cambiando la manera en la que veo la situación en la que me encuentro]

9. When I am feeling negative emotions, I make sure not to express them [Cuando siento emociones desagradables, me aseguro de NO expresarlas]

10. When I want to feel less negative emotion, I change the way I'm thinking about the situation [Cuando quiero sentir menos emociones desagradables, cambio la manera de ver la situación]

Scoring:

Cognitive Reappraisal: Items 1, 3, 5, 7, 8, 10.

Expressive Suppression: Items 2, 4, 6, 9
